# Scaling malaria interventions: bottlenecks to malaria elimination

**DOI:** 10.1136/bmjgh-2023-013378

**Published:** 2023-11-10

**Authors:** Wenhui Mao, Rianna Cooke, Diana Silimperi, Elina Urli Hodges, Ernesto Ortiz, Krishna Udayakumar

**Affiliations:** 1 Duke Global Health Innovation Center, Duke University, Durham, North Carolina, USA; 2 Innovations in Healthcare, Durham, North Carolina, USA

**Keywords:** Malaria

## Abstract

The slow progress in malaria control efforts and increasing challenges have prompted a need to accelerate the research and development (R&D), launch and scaling of effective interventions for malaria elimination. This research, including desk research and key informant interviews, identified the following challenges along the end-to-end scale-up pathway of malaria interventions. Underinvestment in malaria R&D persists, and developers from low-resource settings are not commonly included in the R&D process. Unpredictable or unclear regulatory and policy pathways have been a hurdle. The private sector has not been fully engaged, which results in a less competitive market with few manufacturers, and consequently, a low supply of products. Persistent challenges also exist in the scaling of malaria interventions, such as the fragmentation of malaria programmes. Further efforts are needed to: (1) Strengthen coordination among stakeholders and especially the private sector to inform decisions and mobilise resources. (2) Increase engagement of national stakeholders, particularly those in low-income and middle-income countries, in planning for and implementing R&D, launching and scaling proven malaria interventions. (3) Use financial incentives and other market-shaping strategies to encourage R&D for innovative malaria products and improve existing interventions. (4) Streamline and improve transparency of WHO’s prequalification and guidelines processes to provide timely technical advice and strategies for different settings. (5) Increase effort to integrate malaria services into the broader primary healthcare system. (6) Generate evidence to inform policies on improving access to malaria interventions.

**Summary box**

- Despite rapid progress since 2000s, malaria remains a significant global health challenge, disproportionately affecting low- and middle-income countries (LMICs) and the ambition to eradicate malaria has been met with considerable challenges such as drug resistance, limited access to healthcare in remote and vulnerable populations and climate change.
- Multiple barriers persists along the end-to-end scale up pathway of malaria interventions including underinvestment in malaria research and development, unpredictable or unclear regulatory and policy pathways, lack of engagement with LMICs stakeholders and private sector, and the fragmentation of malaria programs.
- Further efforts are needed to enhance coordination and multi-sector collaboration, empower national stakeholders, leverage incentives and effective market shaping strategies, optimize regulatory and guideline processes, increase effort to integrate malaria services into the broader primary healthcare system, and generate evidence to inform policy design and implementation on improving access to malaria interventions.

## Introduction

Malaria is a significant global health challenge, disproportionately affecting low-income and middle-income countries (LMICs) with weak health systems, limited resources and elevated poverty levels.^1 2^ Several proven prevention and control measures, including insecticide-treated bed nets (ITNs), indoor residual spraying (IRS), rapid diagnostic tests and antimalarial treatments, have contributed to the reduction of the malaria burden.^1^ However, significant and growing coverage gaps persist in these malaria interventions. By 2020, 65% of households in sub-Saharan Africa possessed at least one ITN, but overall access to and use of ITNs have been on decline since 2017.^1^ This decrease in access is not confined to ITNs. Back in 2010, 5.8% of the population at risk were protected by IRS, but by 2020, only 2.6% had such access. Despite WHO’s long-standing endorsement of artemisinin-based combination treatment (ACTs) and intermittent preventative therapy in pregnancy, the actual delivery of these treatments to patients in need remains alarmingly inadequate (approximately 20% for each intervention).^3 4^

Numerous organisations or initiatives play a vital role in supporting malaria control efforts. The Global Fund to Fight AIDS, Tuberculosis and Malaria (Global Fund), for instance, contributes 63% of all international financing for malaria programmes.^5^ Despite these efforts, financing for malaria programmes remains insufficient. In 2020, the total funding for malaria fell significantly short, reaching only half of the US$6.8 billion annual goal necessary to propel the world towards malaria elimination.^6^

Despite substantial progress achieved since 2000, the Global Technical Strategy for Malaria 2016–2030, the Sustainable Development Goals 2025 and 2030 targets for malaria morbidity and mortality are currently off-track. Urgent actions are needed to enhance equitable access to malaria interventions.^7^

**Figure 1:**
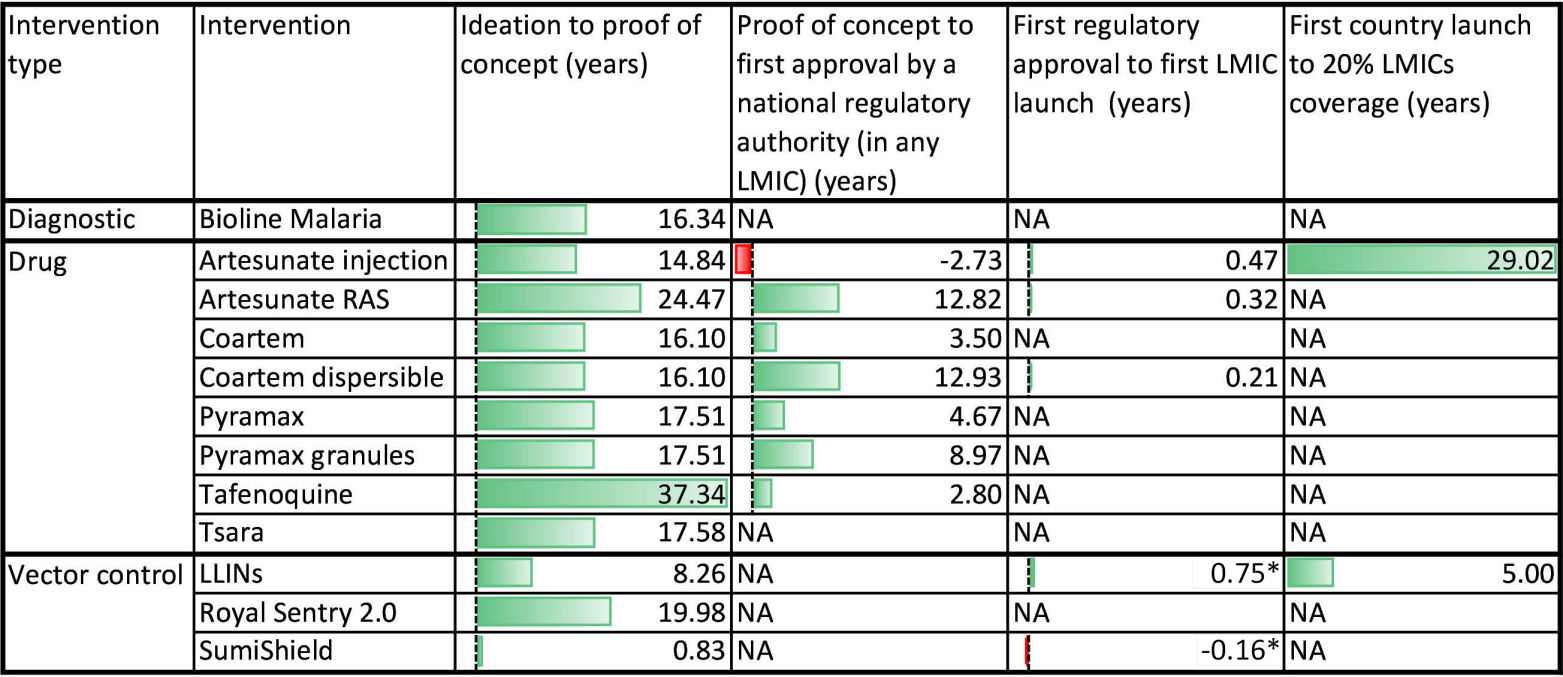
R&D, launch and scale up pathways of selected malaria interventions. LMIC, low-income and middle-income country; LLIN. long-lasting insecticidal nets; long-lasting insecticidal nets; RAS, rectal artesunate. *LLIN and SumiShield used the time between proof of concept to first country launch as proxy for proof of concept to first country launch.

The ambition to eradicate malaria has been met with considerable challenges such as drug resistance, insecticide resistance and limited access to healthcare in remote and vulnerable populations. Furthermore, existing malaria interventions have not effectively scaled, resulting in low access for the populations who need them the most.^8^ Prior efforts through the Launch and Scale Speedometer (Speedometer), an initiative led by the Duke Global Health Innovation Center to strengthen evidence for the development and scaling of global health interventions,^9^ revealed significant data gaps in measuring the introduction and uptake pathways for malaria interventions. However, data from a subset of malaria interventions showed that the journey from ideation to proof of concept took an average of 20 years. Alarmingly, only 2 out of 12 malaria interventions reviewed have achieved a coverage of at least 20% of the target population in LMICs (figure 1). There is a limitation that this finding is based on very limited publicly available data.

The slow progress in malaria control endeavours underscores the urgency to expedite the research and development (R&D), launch and scaling of effective interventions for malaria elimination. This includes exploring novel tools and strategies for malaria prevention, diagnosis and treatment. Additionally, it entails the rapidly scaling of proven malaria interventions by enhancing regulatory processes, optimising financing mechanisms and improving service delivery to ensure accessibility for the population in need. This research, consisting of desk review and key informant interviews, aims to identify the challenges along the entire end-to-end scale-up pathway of malaria interventions and proposes recommendations to accelerate access to malaria interventions.

## Multiple barriers in advancing malaria R&D

Current interventions for vector control, prevention and treatment exhibit potential for enhancement in both quality and delivery modalities.^10^ These improvements could effectively tackle administrative and logistical barriers, thereby amplifying the uptake of interventions in resource-constrained settings. Examples of potential improvements include the development of durable, high-quality bed nets, streamlining vaccine regimens into simpler two-dose or even single-dose schedules and advancing rapid diagnostic tests with higher accuracy to enable early treatment initiation.^11 12^ Regrettably, there is a dearth of adequate attention and funding directed towards driving these critical improvements.

The urgency for R&D of innovative interventions cannot be overstated, particularly to counter emerging threats and advance toward malaria elimination within the coming decade. An alarming instance of this urgency is the evolving behaviour of vectors, demonstrating resistance to existing insecticides and shifting their activity to daytime.^13^ Additionally, drug resistance to ACT treatment has been reported in various locations, highlighting the pressing need for novel antimalarial solutions.^14^ Rapid climate change further brings uncertainty, altering the malaria epidemiology. Unfortunately, a significant shortfall in investment dedicated to malaria R&D persists, including focused research on *Plasmodium vivax*, a parasite species that is progressively contributing to the overall malaria burden.^6^

Third, several promising candidates are in the pipeline for both treatment and diagnosis of malaria. However, a significant bottleneck lies in enhancing their operational feasibility, including stabilising the active ingredients within the constraints of low-resource setting, while simultaneously reducing the unit cost for mass production. A potential solution could be the involvement of developers from low-resource settings, who possess valuable experience in adapting products to suit local markets. Their inclusion could play a pivotal role in overcoming such operational challenges. Unfortunately, local manufacturers are not commonly engaged in the R&D process.^15^

The present analysis has pinpointed several underlying causes contributing to the stalling progress in malaria R&D. First, although considerable emphasis is placed on reducing intervention cost by major procurers, commensurate efforts towards enhancing quality and cost-effectiveness have been lacking. This disparity has discouraged investment in quality improvements and innovations.^16^ Second, the absence of a sustainable financing mechanism that incentivizes various stakeholders remains a critical issue. Malaria predominantly affects low-resource settings, offering limited financial incentive for pharmaceutical companies to invest in malaria R&D, given the marginal return on investment^10^ or the perceived small ‘addressable market size’.^6 17^ Moreover, the existing funding mechanisms for innovation tend to favour entrepreneurs in high-income countries with dedicated teams for grant sourcing and alternative funding sources for research, development and scaling.^17^ This leaves stakeholders from LMICs with insufficient involvement, particularly in determining priority areas for funding that require innovation. Finally, coordination mechanisms, coupled with a robust real-time evidence base to identify pressing malaria R&D needs and to optimise R&D decisions, are notably lacking.^16^ A comprehensive road map delineating R&D needs, requisite resources and the timeline for each stage across different product types could significantly improve the resource allocation and coordination efforts in malaria R&D.^18^

## Regulatory and implementation hurdles for malaria interventions

Prior work has underscored the challenges posed by unpredictable or unclear regulatory and policy pathways in malaria interventions. These hurdles impede developers, funders, implementation partners and policy-makers in their efforts to plan, coordinate and mobilise resources for the launching and implementing of critical malaria interventions such as drugs, vaccines and vector control products.^6 19^ For diseases such as malaria that are heavily funded by donors and predominately affecting LMICs which possess limited technical and regulatory capacity, the WHO’s guidelines and prequalification (PQ) activities play pivotal roles in providing essential technical guidance and influencing access to vital malaria interventions. Even with the establishment of the Africa Medicines Agency and measures aimed at strengthening national regulatory agencies worldwide, the WHO PQ system is projected to remain indispensable for many years to come.

The WHO guidelines for malaria present evidence-based recommendations tailored to clinical practice, public health and health policy. These guidelines aim to assist policy-makers and malaria programme managers in endemic countries to formulate national policies and plans tailored to their specific local context. In parallel, WHO PQ endeavours to uphold global standards of quality, safety and efficacy for various interventions, including diagnostics, medicines, vaccines and vector control products, facilitating procurement by multilateral agencies and national governments.^6 20 21^ Typically, PQ of an intervention is contingent on the publication of relevant WHO guidelines or recommendations for the intervention. This reliance requires stronger communication and coordination among various WHO units than has historically been in place, as noted by a recent Speedometer analysis.^19^ The analysis also pointed out the variability in the requirement of guidelines prior to WHO PQ, highlighting a lack of clarity in the WHO PQ and guidelines processes within the institution. Such inconsistencies can contribute to delays in delivering interventions to countries in dire need.

Furthermore, two recent Speedometer case studies found that the WHO’s at-times lengthy timelines for updating guidelines or recommendations appeared to contribute to significant scaling delays, in some cases of multiple years.^17 19^ However, there is limited publicly available data to comprehensively quantify the impact of the delays of WHO guidelines and PQ processes. It would be beneficial for the WHO to provide more transparency and clarity on its regulatory and policy-making processes, including associated timelines. Additionally, while WHO guidelines provide technical recommendations for each intervention and for different purposes (diagnosing, treating, etc), there is an opportunity to further enrich these guidelines by addressing a spectrum of strategies and optimal timing for implementing specific interventions, taking into consideration the local context.^20^

Moreover, the redundancy in both national regulatory and WHO PQ dossier requirements, such as mandating multiple bioequivalence studies, poses additional cost on suppliers and could potentially delay access.^19^ This inefficiency could further discourage suppliers and lead to a less competitive market, placing the quality, price and availability of malaria products at risk in LMICs. Although WHO PQ offers the potential of a vital quality standard for purchasers and development partners, it cannot entirely replace the distinct role of national regulators in reviewing and authorising products for in-country use. However, a crucial need exists for improved coordination between WHO PQ, WHO’s regulatory systems strengthening efforts and national regulatory bodies. The recent announcement of the new electronic PQ system, set to launch in 2024, may create efficiencies and provide greater visibility into the PQ pipeline for all stakeholders.^22^ Strategically, national regulatory entities should consider to developing a fast-track assessment mechanism facilitating rapid review and approval for products cleared through WHO PQ to minimise duplicative efforts, accelerate product accessibility and free up or redirect national regulatory capacities towards evaluating other essential products. Malaria intervention developers with certain prequalified products may also choose to participate in the WHO’s Collaborative Registration Procedure, for which countries have agreed to accelerate registration to speed access.^23^ Lastly, there is a fundamental need to strengthen the capacity at both WHO PQ and national regulatory entities to ensure effective and efficient regulatory support and oversight.

To ensure a stable and accessible supply, the private sector must have strong incentives to manufacture malaria products. When more products become available, the competition among manufacturers can drive the production of higher quality products at reduced costs.^24^ Unfortunately, such a favourable competitive landscape is not a common feature within the malaria product market. Global or multinational manufacturers have little motivation to share intellectual property or transfer technology to local LMIC-based manufacturers. This absence of incentives frequently results in a limited number of manufacturers, which could increase the risk of more consolidated, less available supplies of products. Innovative ways, offering financial or other incentives, should be considered to effectively engage with private actors.

More insights into market shaping and demand forecasting would foster more effective markets. At global and regional levels, there is a scarcity of awareness regarding existing suppliers and the potential scope of demand.^24^ The full manufacturing and supply capacity of public and private actors is often not well understood, representing a missed opportunity to cultivate a competitive and robust market, inclusive of both public and private suppliers. At the national level, limited capacity and resources for monitoring and predicting malaria outbreaks also contribute to a low and unstable demand for malaria interventions.^25^ The resulting mismatch between supply and demand cycles increases the cost and uncertainty for manufacturers, further discouraging their active participation in the malaria market.^26^

## Program fragmentation and insufficient integration of malaria services within health systems

Many enduring challenges exist in scaling malaria interventions and ensuring target populations have access to new or enhanced interventions.^6^ One prominent challenge is the fragmentation observed within malaria programmes. Countries have national plans and adhere to specific budget cycles, while development partners operate on their own agenda and funding cycles. Unfortunately, these timelines and priorities may not always align with the national needs or cycles.^27^ Consequently, misalignment can lead to duplication of efforts in some areas, while other significant issues might be neglected.^28^

The fragmentation of malaria programmes also poses substantial challenges and complexities in information dissemination and management. Various organisations often develop and distribute information on malaria prevention, diagnosis and treatment independently. As a result, policy-makers find it hard to obtain a comprehensive understanding of the malaria epidemic, the existing service provision and to identify gaps and needs in service delivery. More coordination could help improve the cohesiveness and continuity within malaria programmes, both at national and international levels.

With the advent of new malaria interventions, there are growing challenges for health systems to ensure that the drugs, diagnostics, vaccines and vector control products are designed for the specific conditions they are meant to address; reach the right place, at the right time, in the right quantities; and are delivered in a timely and integrated manner. But research focusing on implementation only accounted for 18% of total malaria funding in 2016, with health systems research comprising only 0.4%.^6^ More evidence is needed to support national and subnational policy-makers to effectively design the policies that support malaria programmes. This includes understanding how to apply a blend of interventions that align with specific regional needs, how to prioritise interventions when faced with budget constraints, how to mitigate associated risks and barriers and how to identify and reach the hard-to-reach populations.

While the private sector plays an important role in delivering health services in LMICs,^24^ there have been limited efforts to engage the private sector in scaling malaria interventions. A prime example is the procurement of medical commodities through price negotiation, primarily benefiting the public sector and its facilities. This leaves individuals relying on the private sector for their healthcare paying higher prices (sometimes exorbitantly higher) for these interventions.^17 18^

There is also a growing effort to integrate malaria services within the broader primary healthcare system. For example, malaria services have been fully integrated into the care algorithm, Integrated Management of Childhood Illness (IMCI). The successful scaling of IMCI in health facilities has demonstrated a reduction in child mortality and has been adopted by over 100 countries since 1990s.^29^ For new malaria interventions, such as malaria vaccines, comprehensive planning for their integration within diverse service packages will be critical and needs to address financial resources, workforce and information systems to truly increase access. Thus, in order to maximise uptake of malaria interventions at points of care, integration needs to occur at programmatic level across diverse structures within a Ministry of Health such as infectious disease, primary healthcare or reproductive and maternal child health services. However, currently there is insufficient evidence on the value and effectiveness of integration, including its potential impact on quality and delivery of other interventions ‘packaged’ with malaria services. Moreover, the vital resources to implement such integration at scale are currently lacking.

## Actionable recommendations to make malaria interventions accessible

Progress has been made in malaria control, but much needs to be done to elevate access to malaria interventions and effectively meet malaria-related goals in the coming decade. The growing challenges from climate change and insecticide resistance also call for urgent actions. We propose the following actionable recommendations:

- *Enhance coordination and multisector collaboration*. Building on strong multilateral and bilateral programmes, further opportunities exist to strengthen coordination among development partners, technical organisations, policy-makers and especially the private sector, particularly in LMICs. This concerted effort will inform decisions, mobilise resources and facilitate multisector partnerships and collaborations, catalysing attention and improving access to malaria interventions.
- *Empower national stakeholders*. Increase the involvement of national stakeholders, particularly those in LMICs, in planning for and implementing R&D, launching and scaling proven malaria interventions across all programmatic elements from prevention to treatment. This engagement should be supported by enhanced capacities in disease monitoring and forecasting demand for malaria interventions to enable data driven decision-making at national and subnational levels.
- *Leverage incentives and effective market shaping strategies*. Use financial incentives and other market-shaping interventions as levers to encourage R&D for innovative malaria products and improve existing malaria interventions. Strategies such as product development partnerships, advance market commitments and demand forecasting could be considered to attract more suppliers to measure, grow and address market demand with affordable products and services.
- *Optimise regulatory and guideline processes*. Streamline and improve transparency in the WHO’s PQ and guidelines processes to provide timely technical advice and strategies to drive malaria elimination in diverse settings. Increasing transparency and communication, sharing evidence and expertise among stakeholders may accelerate the process.
- *Increase effort to integrate malaria services into the broader primary healthcare system,* including budgetary process, financial resources and information systems for better planning. Integrated services, including new malaria interventions such as the malaria vaccine, will reach more populations in need.
- *Generate evidence to inform policy design and implementation on improving access to malaria interventions,* including evidence that tracks the timelines, identifies barriers and bottlenecks in the launching and scaling of malaria interventions, as well as learnings from implementing malaria programmes at national and subnational levels.

## Data Availability

Data are available in a public, open access repository.
